# High post-anthesis temperature effects on bread wheat (*Triticum aestivum L.*) grain transcriptome during early grain-filling

**DOI:** 10.1186/s12870-020-02375-7

**Published:** 2020-04-16

**Authors:** Richard I. Kino, Till K. Pellny, Rowan A. C. Mitchell, Asier Gonzalez-Uriarte, Paola Tosi

**Affiliations:** 1 School of Agriculture Policy and Development, University of Reading, Whiteknights, PO Box 237, Reading, RG6 6AR UK; 2 Rothamsted Research, West Common, Harpenden, Hertfordshire, AL5 2JQ UK; 3 Current affiliation: European Bioinformatics Institute, Wellcome Genome Campus, Cambridgeshire, CB10 1SD UK

**Keywords:** RNA-Seq, Wheat, Grain development, Post-anthesis, Temperature, Cell wall, Pericarp, Transcriptomics

## Abstract

**Background:**

High post-anthesis (p.a) temperatures reduce mature grain weights in wheat and other cereals. However, the causes of this reduction are not entirely known. Control of grain expansion by the maternally derived pericarp of the grain has previously been suggested, although this interaction has not been investigated under high p.a. temperatures. Down-regulation of pericarp localised genes that regulate cell wall expansion under high p.a. temperatures may limit expansion of the encapsulated endosperm due to a loss of plasticity in the pericarp, reducing mature grain weight. Here the effect of high p.a. temperatures on the transcriptome of the pericarp and endosperm of the wheat grain during early grain-filling was investigated via RNA-Seq and is discussed alongside grain moisture dynamics during early grain development and mature grain weight.

**Results:**

High p.a. temperatures applied from 6-days after anthesis (daa) and until 18daa reduced the grain’s ability to accumulate water, with total grain moisture and percentage grain moisture content being significantly reduced from 14daa onwards. Mature grain weight was also significantly reduced by the same high p.a. temperatures applied from 6daa for 4-days or more, in a separate experiment. Comparison of our RNA-Seq data from whole grains, with existing data sets from isolated pericarp and endosperm tissues enabled the identification of subsets of genes whose expression was significantly affected by high p.a. temperature and predominantly expressed in either tissue. Hierarchical clustering and gene ontology analysis resulted in the identification of a number of genes implicated in the regulation of cell wall expansion, predominantly expressed in the pericarp and significantly down-regulated under high p.a. temperatures, including endoglucanase, xyloglucan endotransglycosylases and a β-expansin. An over-representation of genes involved in the ‘cuticle development’ functional pathway that were expressed in the pericarp and affected by high p.a. temperatures was also observed.

**Conclusions:**

High p.a. temperature induced down-regulation of genes involved in regulating pericarp cell wall expansion. This concomitant down-regulation with a reduction in total grain moisture content and grain weight following the same treatment period, adds support to the theory that high p.a. temperatures may cause a reduction in mature grain weight as result of decreased pericarp cell wall expansion.

## Background

Increases in average global temperatures of between 0.35–0.7 °C by the year 2035 have been predicted and are expected to be accompanied by an increase in the frequency and severity of extreme high temperature events such as heatwaves and exceptionally hot days [1]. High temperatures (> ~ 28–30 °C [2]), when experienced post-anthesis (p.a.) can significantly reduce mature grain weight in wheat, consequently reducing yields [3–6].

Several physiological responses have been identified as causal to the reduction in wheat grain weight observed following high p.a. temperature exposure and they mainly relate to source limiting factors such as reduced chlorophyll content [7], reduced root biomass [8] and reduced mobilization of photoassimilates [9]. However, it has also been suggested that exposure to high p.a. temperatures may induce cell wall modifications within the developing wheat grain affecting the sink capacity of the grain and contributing to the observed reduction in grain weight [10, 11].

Cereal grain growth requires both the endosperm and the maternally derived tissue that surrounds it, i.e. the pericarp, to expand during grain filling, and it has been suggested that the pericarp may exert physical control over the size of the wheat grain endosperm by allowing or containing its expansion [10, 12, 13]. This phenomenon, in turn, would be regulated by the potential and ability for expansion of pericarp cell walls. The expression pattern of gene transcripts with a high degree of homology to a cell wall modifying protein in wheat grain, α-expansin (*pTaExpA6),* appear to corroborate this hypothesis: the expression of these genes peaked at around 2-6daa in the pericarp prior to the genes being expressed also in the endosperm [10]. Further evidence of the role played by the pericarp on grain size potential is the identification in a double haploid wheat mapping population of a robust QTL for increased pericarp cell length, associated with longer grains and a 6.9% increase in grain weight [13]. In optimal growing conditions (12–21 °C for wheat [14, 15]) the process of grain expansion is highly coordinated between the pericarp and endosperm [16, 17], however, the interaction between the two tissues has not been investigated in depth under high p.a. temperatures. Previous studies investigating the effect of high temperature on maternal tissues have focused on high temperatures during the pre-anthesis period and, therefore, on their impact on the size of the maternal carpels. High temperature was observed to induce a reduction in size of the carpels with a correlation between carpel size pre-anthesis and final grain weight having also being reported [18]. A number of cell wall modifying proteins have been identified in cereal grains as putatively facilitating the expansion of the different tissues of the grain during early grain development, including expansins (EXPs) [10] and xyloglucan endotransglucosylase/hydrolases (XTHs) [19]. However, the expression patterns of these proteins within the different wheat grain layers under high p.a. temperatures is unknown. Global transcriptomic datasets from RNA-Seq and microarray profiling of wheat grain, both at the whole grain [20] and individual tissue scale [21–23] are now publicly available. One of these transcriptomic studies included an analysis of grain grown under high temperature which showed that increased temperature resulted in an acceleration of the normal trends in transcriptomic expression [20], which is consistent with an overall acceleration of grain development observed under high temperature regimes. In order to investigate whether high p.a. temperatures reduce grain weight by causing a premature decrease in the ability of the cell walls of the pericarp to expand during development with respect to the endosperm, analysis of the effect of high p.a. temperature on the transcriptome of these tissues is required.

In this study, we compared RNA-Seq data obtained from whole grains that underwent a p.a. high temperature treatment against existing sequence data from individual pericarp and endosperm tissue, with the aim to identify genes predominantly expressed in these tissues and whose expression patterns are significantly affected by temperature. In addition, the effect of high p.a. temperatures on mature grain weight, and grain moisture content dynamics was also investigated due to the close association between grain moisture content and shifts in transcript expression [20], and between maximum grain moisture content and final grain weight [24].

## Results

Two separate experiments were performed, the first (Experiment 1) aimed at determining the specific parameters, i.e. temperature and the length of application of this temperature, that induced a heat stress response in the specific wheat cultivar used, measurable as a reduction in mature grain weight. The second experiment (Experiment 2) aimed at investigating the effect of high p.a. temperature on grain moisture dynamics and on the transcriptomic profiles of the pericarp and endosperm.

### Mature grain dry weight and grain yield

**Fig. 1:**
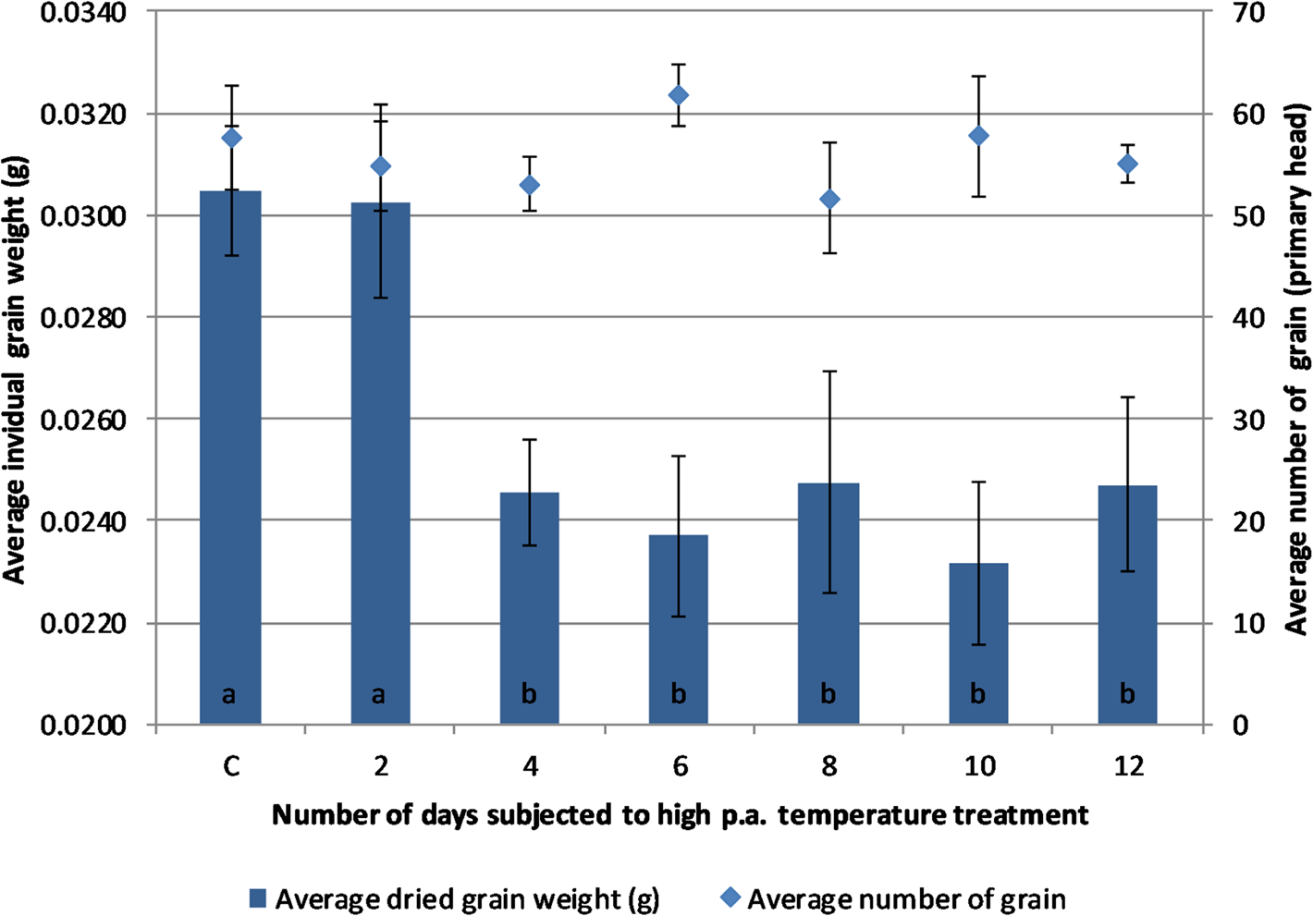
Average mature dried grain weight and grain number from primary tillers exposed to high p.a. temperature treatment (35 °C) for different lengths (number of days) from 6daa. Error bars represent the standard error of the means of 8 biological replicates. The ‘C’ label on the x-axis represents the control/no-treatment group. Labelling with different letters indicates significant differences in grain weight between groups according to the LSDs between the means at *p* < 0.05. Treatments with the same letter had no significant difference between them

Average mature grain weight in primary tillers was significantly reduced in Experiment 1 by exposure to high p.a. temperature treatments for durations of 4-days or more based on comparison of the least significant differences (LSDs) of the means against the overall mean for each treatment (ANOVA, df = 6, f = 3.54, *p* = < 0.05). However, there was no significant difference between the control treatment group and those subjected to 2-days of high p.a. temperature (Fig. 1).

On a whole plant basis (see “Additional file 1”) there was no significant difference between treatments in mature grain number; however, high p.a. temperature treatment over 4 days length generally produced significantly lower average mature grain weights (ANOVA, df = 6, f = 6.89, *p* = < 0.001), and therefore, lower grain yield (the exception being the 12-days long treatment, for which no statistically significant difference in grain yield was observed).

### Grain moisture content and dry matter accumulation dynamics

**Fig. 2:**
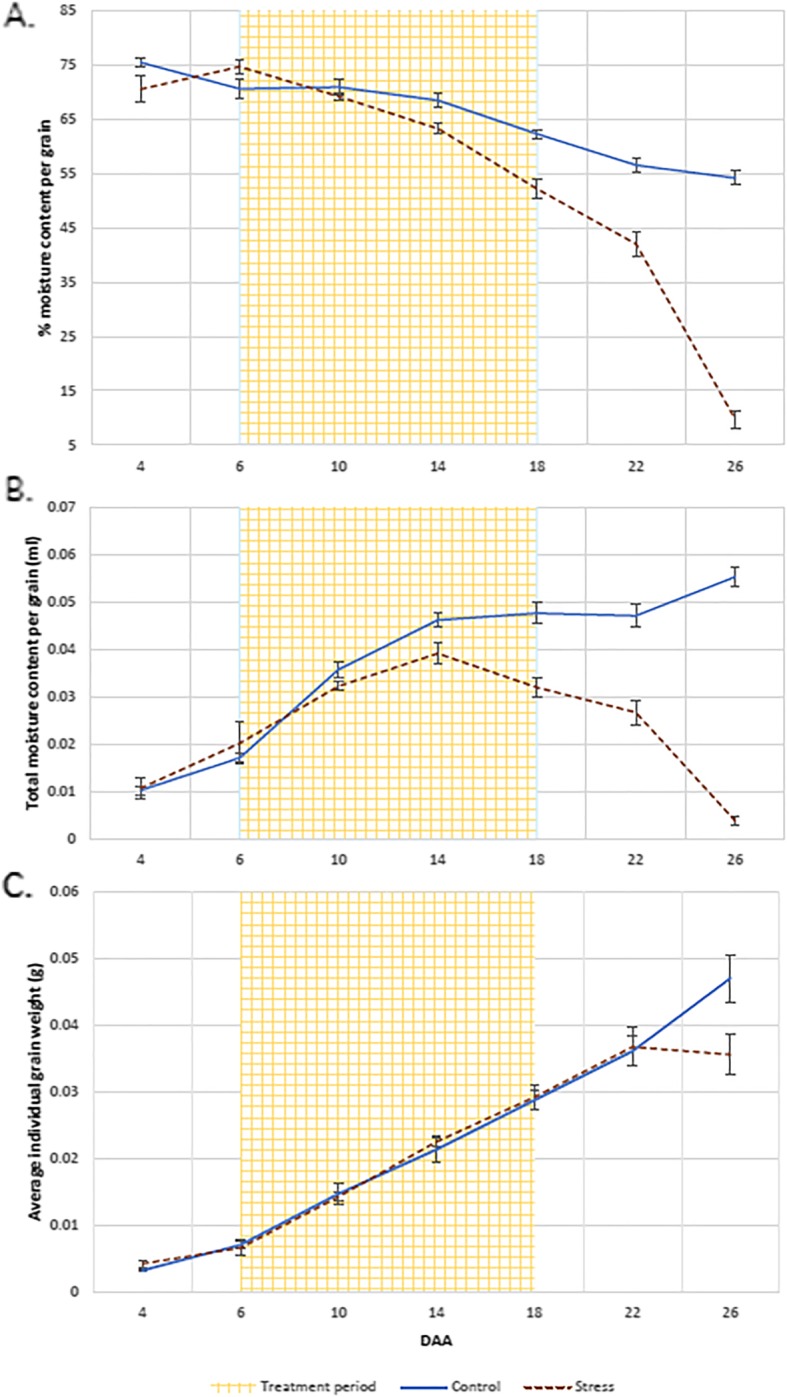
Average percentage moisture content (**a**), total moisture content (**b**), and dry weight (**c**) of grains (*n* = 16) collected from primary tillers exposed to high p.a. temperature treatment from 6daa until 18daa. Solid lines represent data for control grains whilst dotted lines represent data for heat treated grain. Error bars represent the standard error of the mean of 4 biological replicates

Average percentage grain moisture content^.^ was significantly affected by high p.a. temperature treatment (ANOVA, df = 1, f = 1048.70, *p* = < 0.001) in addition to average total grain moisture content (ANOVA, df = 1, f = 224.80, *p* = < 0.001) in Experiment 2, with a significant interaction effect of daa in both (ANOVA, df = 6, f = 65.53 ^1.^ 34.82 ^2.^*p* = < 0.001). Average percentage grain moisture content (Fig. 2 (a)) and average total moisture content (Fig. 2 (b)) were significantly reduced in high p.a. temperature treated grains from 14daa onwards according to the LSDs of the means. No significant difference was observed in grain filling rate between the two treatments, but the duration of grain filling was shorter in the treated plants (Fig. 2 (c)).

### Wheat grain transcriptomics data analysis

#### RNA-Seq statistics

RNA-Seq transcriptomes were obtained for RNA isolated from whole grain at 6daa (2 reps), 10daa and 14daa (3 reps each) for controls, and at 10daa and 14daa (3 reps each) for high p.a. temperature treated samples. RNA-Seq statistics for the 14 sequenced samples are displayed in “Additional file 2”. The lowest alignment rate was 79.1%, the average alignment rate was 89.68%.

#### Principal component analysis (PCA)

**Fig. 3:**
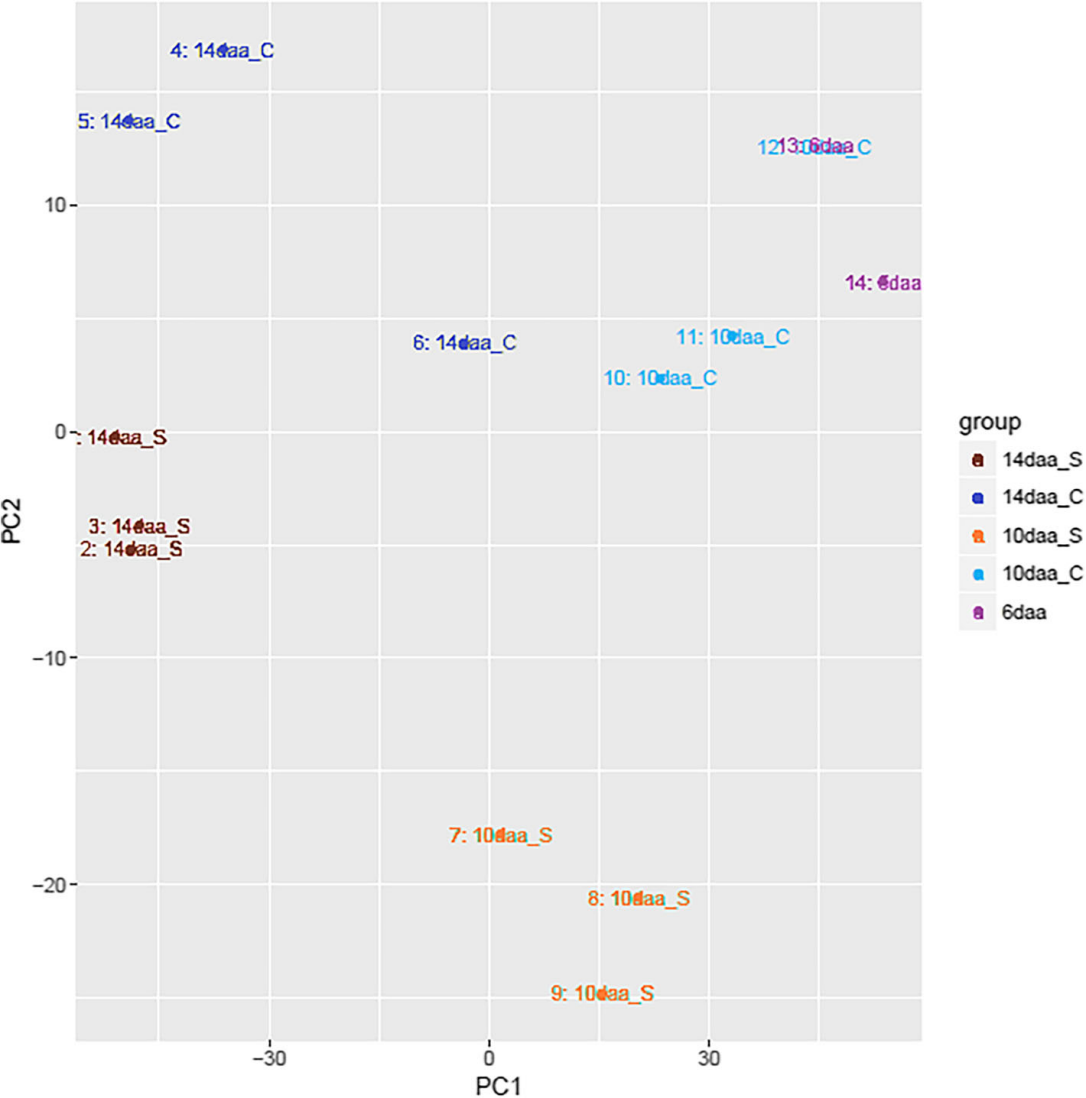
Principal component analysis of the transcript counts for the 14 samples analysed by RNA-SEQ. PC1 on the X axis captures the greatest variation (p.a. developmental stage) and accounts for 78% of the total variance around the PCs whilst PC2 on the Y axis captures the second greatest variation (effect of high p.a. temperature treatment) and accounts for 9% of the total variance around the PCs. Samples with similar transcription patterns group together

The RNA-Seq data set of the 14 samples was subjected to principal component analysis (PCA). The data showed clear grouping of replicate samples for same developmental stage and treatment groups, indicating similar gene expression between samples within treatment groups (Fig. 3). The first principle component (PC1) accounted for 78% of variance in gene expression between samples and separated points mostly by developmental stage (increasing with decreasing PC1). The second principle component (PC2) captures the second greatest variation (effect of high p.a. temperature treatment) and accounted for 9% of the total variance in gene expression between samples. Points for control samples had lower PC1 values the later their daa stage, and treated samples had lower PC1 values than controls, indicating that PC1 is primarily reflecting grain development and that temperature treatment is accelerating development.

#### Differential expression of genes associated with individual grain layers under high p.a. temperature treatment

The availability of existing RNA-Seq data sets from isolated grain tissues from Pearce et al. [23] (RNA isolated from inner and outer layers of pericarp and from endosperm of 12daa grains) and Pellny (*published online*: Arrayexpress - E-MTAB-8397) (RNA isolated from pure starchy endosperm of 17daa grains) allowed for genes predominantly expressed in either the pericarp or the endosperm to be determined. The expression patterns of these genes were then analysed in the RNA-Seq data obtained in this experiment from whole grain. In both the above mentioned tissue-specific studies, the experimental material was grown under similar environmental conditions as ours (e.g. ~ 18 °C) and in the study by Pearce et al. [23] grains were sampled at a stage (~12daa) similar to that used in our whole-grain RNAseq experiment.

Initial analysis of differential gene expression between the pericarp and endosperm RNA-Seq reference data sets [23, Pellny, *published online*, Arrayexpress - E-MTAB-8397] revealed 39,823 DEGs. After cross comparison and filtering, 463 genes were determined to be predominantly expressed in the pericarp whilst 542 were identified as being predominantly expressed in the endosperm (see “Additional files 3 and 4”).

These groups of tissue-specific genes were then compared with a list of DEGs identified between control and high p.a. temperature treatments at 10 and 14daa (4981 and 3652 DEGs respectively) from this same experiment. This enabled the identification of genes present in both sets, that were therefore likely to be predominantly expressed in either the pericarp or endosperm and whose expression was significantly affected by high p.a. temperature at the measured developmental stages. Of the 463 DEGs determined to be predominantly expressed in the pericarp, 166 genes were differentially expressed between treatments at 10daa and 92 differentially expressed at 14daa, with 48 genes being differentially expressed between treatments at both 10daa and 14daa. Out of 542 DEGs predominantly expressed in the endosperm only 7 genes were differentially expressed between treatments at 10daa and 39 at 14daa, with 2 genes being differentially expressed at both stages.

**Fig. 4:**
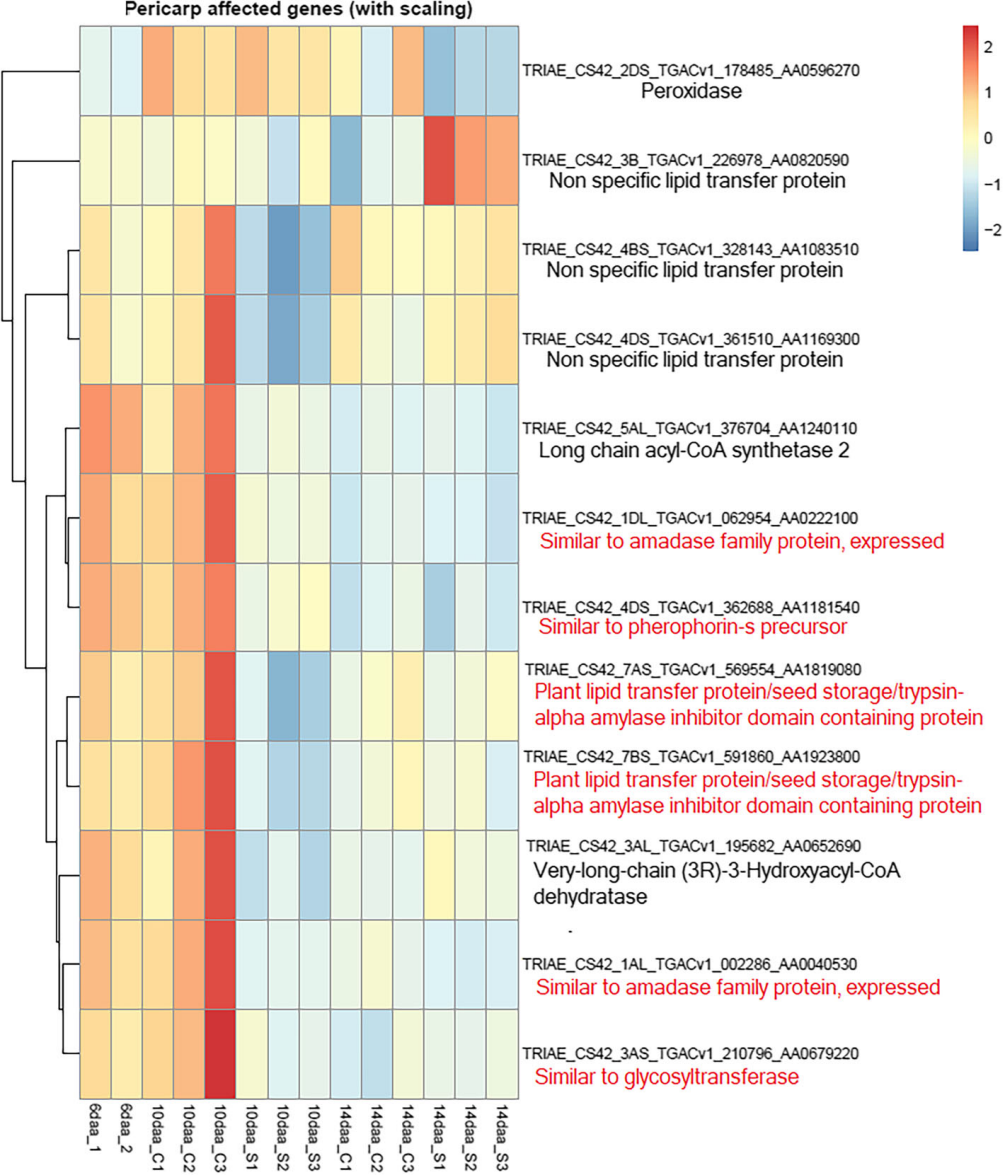
Heat map of significant pericarp predominant DEGs between 10 and 14daa with a significant interaction of high p.a. temperature treatment. Rows are DEGs whilst columns are samples (C = control, S = stressed). Relative abundances have been scaled per row using z-score scaling and clustered globally in order to group genes with similar expression profiles. The scale bar represents fold change in expression based on CPM values. Gene products in red represent available rice (Sativa japonica, *Oryza sativa*) orthologues where gene annotations were not available from the Wheat genome.

The number of DEGs between the two tissues that were significantly differentially expressed between 10 and 14daa with a significant interaction of heat treatment was then determined. DEG analysis between samples at 10 and 14daa performed on RNA-Seq data that had not undergone any tissue-specific filtering revealed 115 DEGs which also presented significant differential expression between high p.a. temperature treated and control samples. When compared against the tissue specific pericarp and endosperm gene lists, 12 genes out of these 115 DEGs were predominantly expressed in the pericarp (Fig. 4), and included genes encoding lipid transfer proteins (LTP) and a peroxidase, while only 1 match was found with the list of predominantly endosperm expressed genes and corresponded to a member of the seed-type vacuolar processing enzyme family.

#### Cluster analysis

**Fig. 5:**
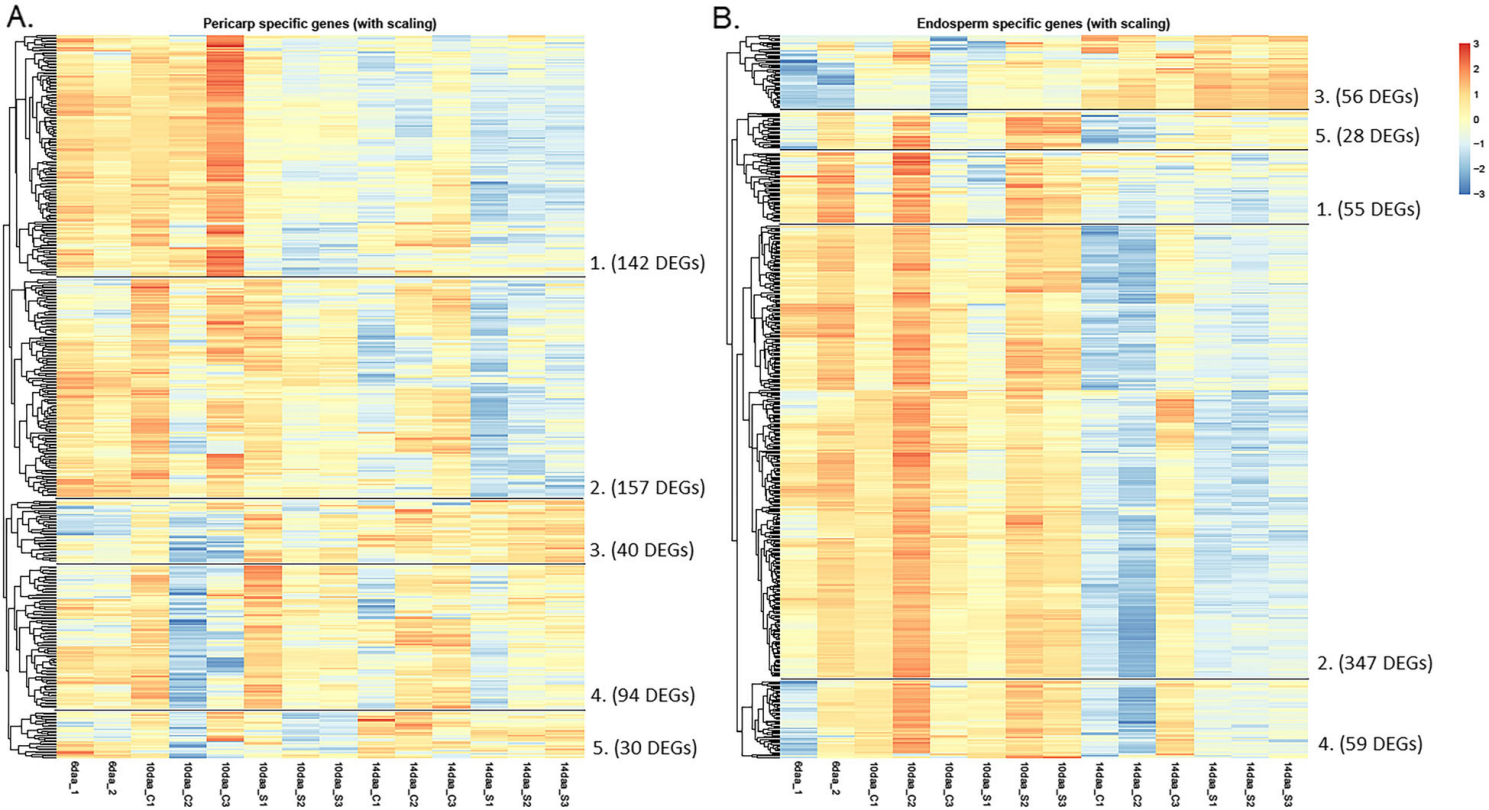
K-means clustering (*n* = 5) of pericarp predominant DEGs (*n* = 463) (**a**) and endosperm predominant DEGs (*n* = 542) (**b**). Relative expression per gene representative of Log-CPM. Numbered sections of the heat-map represent the 5 gene clusters

A k-means clustering algorithm was performed on the 463 pericarp predominant DEGs and 542 endosperm predominant DEGs in order to group these genes in terms of similarity of expression patterns across developmental time points and between control and high p.a. temperature treatments. Therefore, the pericarp and endosperm predominant DEGS were divided into 5 clusters each according to the k-means algorithm (Fig. 5a and b, respectively).

CPM values were collected for each gene from the pericarp and endosperm predominant lists and for each sample from the different developmental stages and treatments. These were then log transformed. The median of the log-CPM values for each of the five clusters from both the pericarp and endosperm predominant genes were calculated in each sample and this value, rather than the mean, was used as measure for gene clusters to avoid a large influence of extreme expression values.

#### Expression of clusters from pericarp and endosperm predominant gene lists

**Fig. 6:**
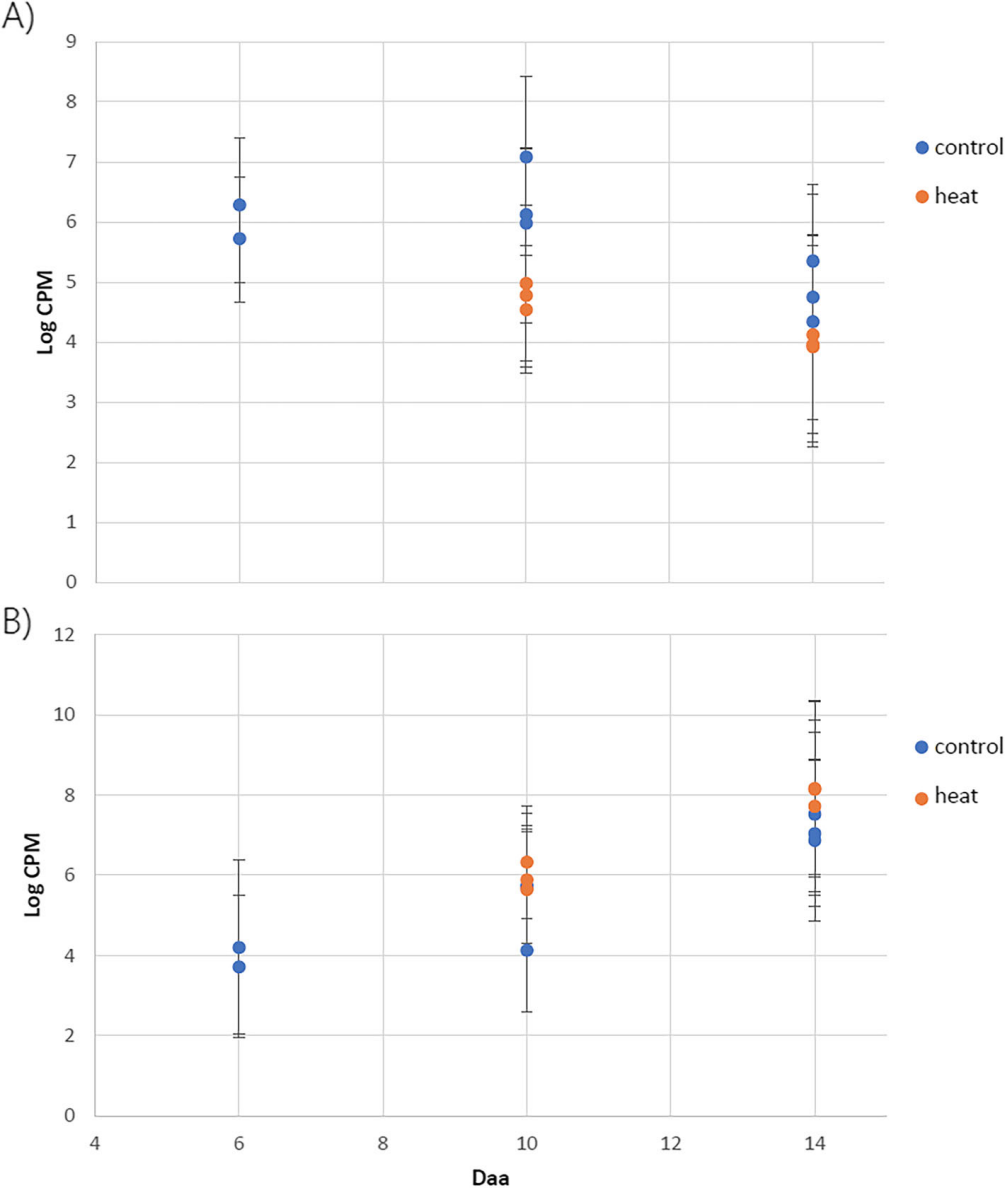
Median Log-CPM values from **a**) pericarp cluster 2 and **b**) endosperm cluster 3, between control and high p.a. temperature treated RNA-SEQ samples at 10 and 14daa and in control treated samples at 6daa. The 6daa stage was included as an early developmental point of reference. (pericarp cluster 2 (*n* = 157), endosperm cluster 3 (*n* = 56). Error bars represent 1 standard error

To test the hypothesis that development is differently affected in pericarp and endosperm by high p.a. temperature, gene expression clusters whose average expression significantly changed between stages were further analysed. The median log-CPM values for each cluster were compared between high p.a. temperature treated and control samples at 10 and 14daa using a 2-way ANOVA. The only pericarp cluster to exhibit significant differences in expression values between control and high p.a. temperature treated samples (2-way ANOVA, f = 26.159, *p* = < 0.001) and between 10 and 14daa (2-way ANOVA, df = 1, f = 24.20 *p* = < 0.001) was pericarp cluster 2, with gene expression within this cluster down-regulated under high p.a. temperature at both 10 and 14daa. However, there was no significant interaction between the treatment and developmental stage on gene expression within the cluster (p= > 0.05) (Fig. 6 (a)).

On the contrary, expression of genes within endosperm cluster 3, the only cluster to exhibit significant differences in expression values between treatment and developmental stages, was significantly up-regulated under high p.a. temperature treatment (2-way ANOVA, df = 1, f = 6.98, p= > 0.001) and between 10 and 14daa (2-way ANOVA, df = 1, f = 42.35, p= > 0.001). However, as observed with pericarp cluster 2, no significant interaction between the developmental stage and the treatment on the gene expression pattern was observed (p= > 0.05) (Fig. 6 (b)). For both of these clusters, the effect of high p.a. temperature can be interpreted as an acceleration of development since it increases expression for pericarp cluster 2 which has a rising trend with development, and decreases it for endosperm cluster 3 which has a falling trend.

#### Gene ontology of significant pericarp and endosperm clusters

The pericarp genes of cluster 2 were cross-referenced in the Knetminer software with the user-specified search term ‘cell wall’. Of the 157 genes within the cluster, 120 of the genes registered a relevance score on the Knetminer knowledge database, with values ranging from 0.05 to 544.88. The genes with the 10 highest relevance scores (highest association with the search term) included three encoding for endoglucanases within the GLU2 family, in addition to two xyloglucan endotransglucosylases, a gene encoding a β-EXP, and an aquaporin (Table 1). Cross referencing of 56 endosperm predominant genes in endosperm cluster 3 with the term ‘cell wall’, returned relevance scores for 43 of the genes; however, values ranged from 0.12 to 39.22, indicating that the strength of the association between endosperm clusters 3 with the search term was lower compared to the pericarp cluster 2 genes.

**Table 1:**
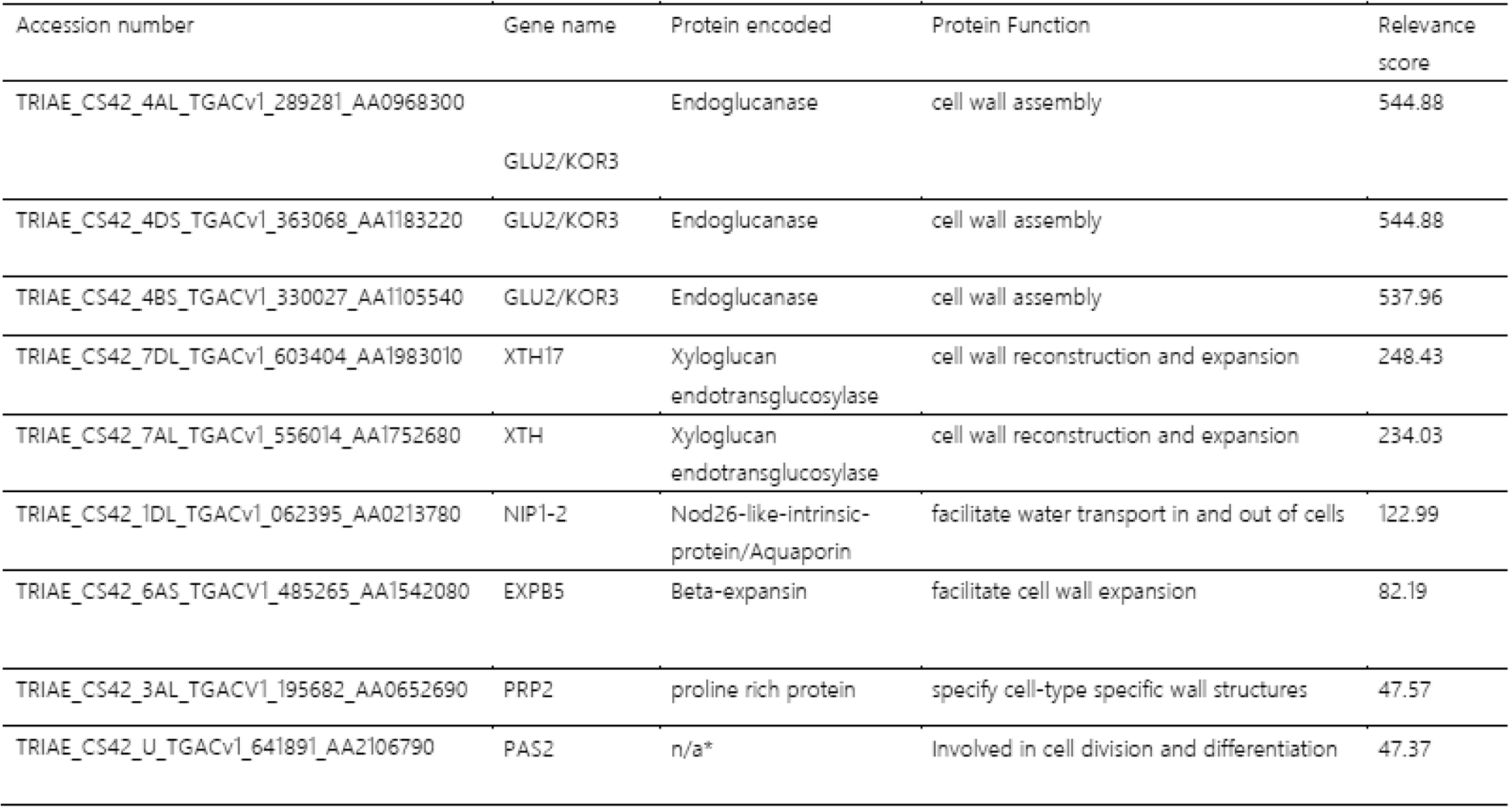
Knetminer output of the ten highest relevance scores of genes from pericarp specific cluster 2 cross-referenced with the search term ‘cell wall’. *Unknown protein encoded.

Gene ontology term enrichment performed on the pericarp cluster 2 and endosperm cluster 3 allowed for the statistical determination of overrepresentation of genes within the sample set, deduced through comparison against all the available potential genes contained within the available reference genome. The analysis then references genes from the sample list against a database of GO-terms that represent a number of biological processes within plant development. In pericarp cluster 2, the biological process with the highest degree of gene over-representation was ‘cuticle development’ (GO id: 0042335), whereas in endosperm cluster 3, genes involved in ‘the killing of cells of other organisms’ (GO id: 0031640) were the most significantly overrepresented genes.

## Discussion

Regulation of wheat grain weight potential by the maternal grain tissues during development has been previously suggested and investigated by other groups [10, 13, 18], but to our knowledge, this work represents the first example of this interaction being explored under high p.a. temperatures. Using RNA-Seq analysis and cross-referencing with existing data sets [23, Pellny *published online*: Arrayexpress - E-MTAB-8397] we were able to better characterise the effect of high p.a. temperatures on the expression profiles of genes predominantly expressed in either the pericarp or endosperm and relate these genes to particular cellular mechanisms. We have focused in particular on genes involved in cell wall restructuring, since our driving hypothesis was that high temperatures, when experienced p.a., would disproportionally accelerate the rate of development of the pericarp tissues compared to the endosperm, resulting in a premature decrease in the ability of pericarp cell walls to expand. This, in turn, would limit endosperm expansion, and therefore grain size.

### High p.a. temperature exposure reduces mature grain weight in cv. Cadenza

The early grain filling phase (6daa-10daa) has been the focus of previous studies investigating the effect of high p.a. temperature on wheat, with high p.a. temperatures during this developmental stage having been reported to result in reductions in mature grain weight [25–29]. The results of Experiment 1, in this study, show that high p.a. temperatures applied towards the end of the celluarisation phase of grain development (around 6daa) for 4-days or longer reduce mature grain weight in wheat, whilst a 2-day high p.a. temperature treatment was insufficient to produce significant differences. This is in contrast with the results of Talukder et al. [29], who observed a reduction in mature grain weights in 4 commercial Australian bread varieties subjected to a single day’s exposure of high p.a. temperature treatment (35 °C) between 7 and 10daa, and highlights the genotypic variability in the response of wheat cultivars to high p.a. temperatures.

The results of Experiment 1 also suggest that there is not a proportional effect between the duration of a high p.a. temperature treatment and the reduction in mature grain weight: the reduction in mature grain weight observed in plants that had undergone 4-days of treatment was not significantly different to those observed in plants that experienced 6, 8, 10 and 12 days of high p.a. temperature treatment. This result supports suggestions by Stone et al. [30] that when brief periods of high p.a. temperature occur, yield potential in wheat is decreased by a more or less a fixed extent. The results of this experiment also support the findings of Nuttall et al. [31] who observed that the application of 4-days of cumulative exposure to a high p.a. temperature treatment of 35/15 °C from 2daa for 6 h per day resulted in 15% reduction in individual grain weight in *cv. Yipti* and that this reduction plateaued from this point onward with further durations of treatment. Therefore, 4-days of high p.a. temperature treatment, either applied consecutively or days apart, would represent a threshold beyond which sensitivity to high p.a. temperature declines, and possibly suggests that the high temperature treatment may trigger the imposition of a structural limit on the sink capacity of the grain.

Although maternal tissue control of seed size and endosperm sink capacity has been well studied [17, 32], the mechanism via which this is achieved has not so far been established. High p.a. temperature treatment, administrated as reported in the experiments here discussed, induced significant down-regulation of pericarp genes with a known role in regulating or modifying cell wall expansion. The down regulation of these genes at around 10daa following the same treatment duration that was demonstrated to result in a reduction in mature grain weight in Experiment 1 adds further support to the hypothesis that high p.a. temperature induces modifications to the expansion capability of the pericarp, which may constrain endosperm cell expansion and present a degree of control over final grain size and weight.

### High p.a. temperatures reduce the ability of the grain to accumulate water

Water accumulation is the main driver for endosperm cell expansion and grain moisture content is therefore closely associated with the developmental stage of the wheat grain [20, 33]. Furthermore, maximum moisture content of the wheat grain has been reported as a reliable indicator of maximum grain volume [34] and, therefore, of final grain weight [35]. Our study showed that high p.a. temperatures affects the potential for water accumulation in the grain: maximum grain moisture content was significantly lower in high p.a. temperature treated grains compared to control grains and this maximum was reached 4-days earlier, at 14daa. Pericarp degeneration, detectable as a loss of greenness, has been previously been shown to occur prior to the termination of rapid net water accumulation in wheat grain [36]. In addition, the degeneration of the pericarp was postulated to be causally related to the achievement of the maximum wheat grain sink capacity, on the basis that the end of endosperm cell division and the cessation of net water accumulation closely coincide [37].

At the transcriptomic level, in this same time interval (6–14 daa), high p.a. temperatures induced down-regulation of genes predominantly expressed in the pericarp and involved in cell wall expansion in grains sampled 10daa, prior to the stabilisation of maximum water content. In support of this observation, Lizana et al. [10] also reported a down-regulation in the expression of expansin transcripts just prior to the stabilisation of grain moisture content. Our result suggests that a premature loss of cell wall plasticity in the pericarp under high p.a. temperature may be at the basis of the reduced volumetric capacity observed in wheat grain exposed to high p.a. temperature.

### Pericarp gene expression under high p.a. temperature

Studies by Altenbach and Kothari [38] and Wan et al. [20], showed that high p.a. temperature accelerate the normal pattern of gene expression within the wheat grain. In our study, where treatment was applied from 6daa, we observed the largest number of DEGs between high p.a. temperature treated and control samples at 10daa, after only 4-days of treatment, rather than at the later time point of 14daa. In agreement with the findings of the above cited authors, PCA showed that high p.a. temperature accelerates gene transcription patterns associated with development, exemplified by lower PC1 values (lower PC1 value = Increased developmental stage) for high p.a. temperature treated samples than control samples. Furthermore, PC2 separated control and high p.a. temperature treated samples along the Y-axis, suggesting that temperature can affect gene expression by more than simply accelerating development e.g. heat stress responses.

Among the DEGs, we identified 12 predominantly pericarp expressed genes showing both significant differences in expression between 10 and 14daa and a significant interaction with heat treatment. These included 5 LTP genes significantly down-regulated at 10daa in samples exposed to high p.a. temperature, with three of these being subsequently up-regulated at 14daa and two remaining at similar expression levels between the two sampled stages. LTPs are a group of highly divergent, small basic proteins involved in cutin development, cell defence, the water permeability of cell membranes and cell wall extension [39–41].

Of the 5 genes encoding LTPs in our data, 3 of those genes encoded non-specific LTPs (nsLTPs), and members of the nsLTP family have been showed to be responsive to one or multiple abiotic stressors (reviewed in Liu et al. [42]), including drought [43–46], salt [44, 45], low temperature [45, 47], and high temperature [45]. Transcript levels for nsLTPs in wheat have been previously shown to increase from around 12daa until the late stages of grain development [48] under high p.a. temperatures (37/28 °C). Also in our study, nsLTPs encoding genes were significantly differentially expressed between developmental stages and showed a significant interaction with temperature. We observed down-regulation of three nsLTPs at 10daa under high p.a. temperatures compared to expression of these genes at 6daa and 10daa under control temperatures, followed by up-regulation at 14daa. We suggest this pattern of expression may reflect the multiple functions of nsLTPs: these genes may be down-regulated under high p.a. temperature conditions at 10daa, reflecting a general reduction in pericarp cell wall extension consistent with the role of nsLTPS as cell wall plasticisers [40], before being upregulated at 14daa in response to high temperatures, possibly to facilitate cuticle development as part of an earlier maturation response of the grain.

### Peroxidase

Another gene differentially expressed between 10 and 14daa with a significant interaction with temperature treatment was PER2/PER42, a gene encoding an un-specified haem-b class III peroxidase and which was down-regulated in high p.a. temperature treated samples at 14daa. Class III peroxidases are secreted glycoproteins known to be involved in a broad range of physiological processes, including regulation of cell elongation and cell wall modification [49, 50]. They are able to promote rigidity of cell walls by forming strong intracellular bonds by oxidation of aromatic cell wall compounds (e.g. lignin), but they can also generate radical oxygen species (ROS) that can break covalent bonds in cell wall polymers such as hydroxyl radical (OH°) [51, 52]. As such, they perform dual functions within plant cell walls, regulating both cell wall stiffening and relaxation. Lipid peroxidation under high temperatures has been reported during the grain-filling stage in wheat, with high temperatures being associated with increase in membrane damage and decrease antioxidant levels [53]. Also in wheat there is evidence of down-regulation of the activity of antioxidant enzymes like peroxidases occurring under high temperatures [54] and exacerbating the accumulation of ROS. The downregulation of the PER2/PER42 gene observed in our study could be linked to ROS accumulation under heat stress, either as a cause or a response.

### Gene ontology and functional analysis

#### Knetminer analysis

Knetminer webtool analysis with the search term ‘cell wall’ applied to the down-regulated genes within pericarp cluster 2 identified endoglucansases, xyloglucanase, an aquaporin and β-EXP. Endoglucanases are a group of membrane-bound enzymes that hydrolyse cell wall polysaccharides with contiguous (1, 4)-β -glycosyl residues in their chain and are implicated in the breakdown of plant cell walls during development [55]. The KORRIGAN (KOR) gene, a specialised member of the endoglucanases, has been shown to be expressed in the expanding cell walls of *A.thaliana* [56], with one member of the KOR family, the KOR3 gene, having been associated with cell wall biosynthesis [57]. In our study, we report the down regulation of three KOR3 genes in the pericarp under high p.a. temperature, which suggests a reduction in cell wall assembly in the outer layers of the grain and, consequently, in their expansion potential.

Within the pericarp cluster 2 genes, the Knetminer analysis webtool also identified members of the XTH family. Some XTHs have been shown to be down-regulated under drought conditions in Barley’s awns [58], however no study has reported so far on the expression of XTH genes in wheat grain under high p.a. temperature. Our data shows that whilst XTHs were down regulated within the pericarp in both control and treated samples between 10 and 14daa, the reduction in the expression of these genes was greater under high p.a. temperatures, which also suggests a reduction in the expansion capacity of the cell walls within the pericarp. Finally, EXPB5 was also identified from the pericarp cluster 2 genes by the Knetminer webtool when searching with the term ‘cell wall’. EXPB5 encodes a β-EXP, belonging to a class of cell wall proteins which have previously been shown to be crucial in regulating cell wall restructuring via the disruption of hydrogen bonds between cellulose microfibrils and cross-linking glycans, thus allowing cellular expansion under turgor driven pressure [59, 60].

Expression of α- EXP genes derived from wheat grain pericarp RNA has been reported to peak between 8-12daa [61] and to rapidly decline after 25daa [10]. Whilst these studies show the importance of α-EXP genes in the early stages of wheat grain development, there is less information regarding the function and presence of β-EXPs in wheat grains in more advanced stages of development.

α and β EXPs make up the two largest families of EXP genes and while they share only around 20% amino-acid identity [60], members from both families appear to destabilise noncovalent load bearing bonds between cellulose microfibrils in plant cell walls [62].

OsEXPB5, a member of the β-EXP family in rice, has been showed to be involved in root hair growth [63]. While there is little information pertaining to the specific action of EXPB5 in wheat grain cell walls, its presence in pericarp cluster 2 suggest that the expression pattern of this cluster may be representative of changes in the cell wall of the pericarp under high p.a. temperatures: down-regulation of EXPB5 between 10 and 14daa is stronger in high p.a. temperature treated samples, suggesting a higher increase in the rigidity of the pericarp cell walls of these grains compared to untreated samples.

#### GO term analysis

GO term enrichment for pericarp cluster 2 revealed that the most significant GO term was ‘cuticle development’ (GO id: 0042335). The cuticle acts primarily as a permeability barrier involved in regulating water loss and protecting plant organs from dirt, bacteria and other microorganisms [64]. During the early stages of anthesis the developing wheat grain contains a number of osmophilic cuticles or cuticular membranes but most of them are gradually removed p.a. by enzymatic degradation [65] so that only two cuticles persist in the developing grain up to maturity: one being found on the outermost layer of the grain, the outer-epidermis, and the other being positioned on the surface of the nucellar epidermis.

The downregulation of genes contained within pericarp cluster 2 at 10daa in high p.a. temperature treated samples compared to control samples indicates a clear effect of high p.a. temperature treatment on the expression of genes involved in cuticle development. Previous studies have shown that the cuticle of wheat leaves and its specific wax composition is important in regulating drought tolerance in different cultivars [66]. The outer-epidermis cuticle of the wheat grain forms from around 7daa and reaches its maximum thickness by 17daa [65], after which it remains structurally unchanged until maturation, apart from becoming appressed to the inner cuticle. Genes involved in the formation of the cuticle are therefore expected to be down-regulated from around 14-17daa as the cuticle ends its development. We observed down-regulation of genes contained within pericarp cluster 2 and annotated as involved in ‘cuticle development’ from 10daa in high p.a. temperature treated samples, which supports the hypothesis of an acceleration in the development of the pericarp under high p.a. temperature. Given the cuticle’s important role in regulating moisture loss in plant tissues, increased expression of genes involved in cuticle development might be expected in order to reduce water losses at high temperature, but in fact the opposite was observed in the form of a more rapid decrease of these transcripts. Possibly this reflects importance of cuticular transpiration in cooling tissues to maintain function.

The most significant GO term derived from the genes contained within endosperm predominant cluster 3 was ‘The killing of cells of other organisms’ (GO id: 0031640). The genes contained within endosperm predominant cluster 3 experienced increased upregulation under high p.a. temperatures compared to the control samples. Previous studies have shown a large degree of functional overlap in defence-associated genes that respond to both biotic and abiotic defence mechanisms [67]. In addition, a transcriptome microarray analysis of grains from a Chinese bread wheat cultivar (Jimai 20), reported in Yu et al. [68] showed that genes associated with defence against both biotic and abiotic stresses were all up-regulated during the grain filling period, particularly between 11-15daa; the results obtained from our experiment, whereby defence associated genes are up-regulated to a high degree between 10 and 14daa in high p.a. temperature treated grain are in agreement with the above mentioned work. Therefore, it appears that high p.a. temperatures accelerate the expression profile of genes involved in cuticle development in the pericarp (down-regulated) and defence genes within the endosperm (up-regulated) between 10 and 14daa.

## Conclusions

The results of the RNA-Seq analysis conducted in this investigation suggest an acceleration of the expression profiles of select groups of genes predominantly expressed in either the endosperm or pericarp. In the pericarp, the expression of genes involved in the formation of the cuticle and associated lipid-binding and transport genes are significantly affected by exposure of grain to high p.a. temperatures with accelerated down-regulation in high p.a. temperature treated samples, suggesting the earlier formation of the cuticle in the outer layers of the wheat grain. The increased up-regulation of genes involved in defence in the endosperm suggests there may be functional overlap between genes that respond to abiotic and biotic stress during wheat grain development and this up regulation would coincide with structural changes to the cell wall. The down-regulation in the pericarp, under high p.a. temperature of genes such as endoglucanases, xyloglucanases EXP and peroxidase suggests a premature loss of plasticity in the pericarp cell walls that would coincide with a reduction in grain moisture content and therefore in the volumetric capacity of the endosperm. The coincident down regulation of these pericarp predominant genes and the earlier attainment of maximum moisture content of grains under high p.a. temperatures lends support to the theory that high p.a. temperature may prematurely reduce plasticity in the outer layers of the grain resulting in a physical restriction on the growth of the endosperm, since moisture content and final grain weight are closely associated. Further transcriptomic analyses from individual grain layers under high p.a. temperature treatment and investigation into the levels of important cellular substrates such as ROS will shed further light on the effect of high p.a. temperature on the regulation of grain development and grain layer expansion.

## Methods

### Plant material growing conditions and experimentation

*Triticum aestivum cv.* Cadenza was used in both Experiment 1 and Experiment 2; seeds for these experiments were purchased from CPB Twyford Ltd., Royston, U.K. In both experiments, seedlings were grown, individually, in 1.5 L pots containing ~ 950 g of a standard soil mix (75% medium grain peat, 12% sterilised loam, 3% medium grade vermiculite 10% sand and 3.5 kg Osmocote plus™ per m^3^) in a glasshouse with additional overhead lighting set to a 16-h photoperiod (5.00–21.00), with the canopy receiving ~ 450 /m2/s Photosynthetically active radiation (PAR). The average relative humidity (RH) of the growing period was 73% for the plants used in Experiment 1 and 67% in Experiment 2.

Four controlled environment growth cabinets (Weiss Technik™ HGC 1514) were used in both experiments, with two cabinets being used for the high p.a. temperature treatment and two for the control treatment in each experiment. All four cabinets were on a 16-h photoperiod (5.00–21.00) with night-time conditions of 70% humidity and 15 °C. In the high p.a. temperature treatment cabinets, during the light-period, RH was set at 50% whilst the temperature increased from the night-time temperature to 35 °C over 4-h between 5.00–9.00 (an increase in temperature of 0.083 °C per minute). There was also a ramping of the temperature at the end of each light-period, with the temperature decreasing from 35 °C to 15 °C between 19.00–21.00 h (a temperature decrease of 0.166 °C per minute). Therefore, the high p.a. temperature treatment (35 °C) was imposed for 10 h per day. The two control cabinets had the same light-period RH as the high p.a temperature treatment cabinets but with a light-period temperature of 18 °C with no ramping of the temperature between night and day. Average amount of light provided was ~ 550 /m2/s PAR at the canopy level in both sets of cabinets.

For the application of the high p.a. temperature treatments, plants were grouped into differently sized cohorts in both experiment 1 and 2, with cohorts consisting of plants that had all reached anthesis on the same day. These cohorts acted as blocks in the experimental design of each experiment (a diagram explaining the experimental design is provided as “Additional file 5”). Anthesis was defined as the moment when the middle third of the ear of the primary tiller of a plant had anthers protruding. In Experiment 1 a randomised block design was used with 8 blocks comprised of 7 plants and 7 treatments. The 7 plants of each block were grown in the glasshouse until 6daa, after which they were transferred into the controlled environment growth cabinets. One plant from each block was transferred to one of the two control treatment cabinets with the remaining 6 plants from each block being transferred as a group to one of the two high p.a. temperature treatment cabinets. These 6 plants in the high temperature treatment cabinets had each been randomly assigned a treatment duration of either 2, 4, 6, 8, 10 or 12-days. Following an assigned high p.a. temperature treatment duration, each plant was then transferred to the control treatment cabinet until the maximum 12-days of high temperature treatment within the block had been completed. Then the whole block was returned to the glasshouse and grown until maturity. Once they had reached maturity, grains were collected for yield data determination.

In Experiment 2, a randomised block design was used, with 4 blocks consisting of 14 plants in total, with 7 plants being used for the high p.a. temperature treatment and 7 being used as the control. In total there were 5 treatments. All plants within each block were grown in the glasshouse until 6daa before being transferred to either the high p.a. temperature treatment cabinets or the control treatment cabinets. Plants within the cohorts had previously been randomly allocated to one of 7 p.a. collection points (4, 6, 10, 14, 18, 22, 26daa). Plants allocated to 4 and 6daa collections were sampled whilst in the glasshouse, before the high temperature was applied, in order to obtain an early development measurement of grain moisture content. Each individual plant was subjected to one treatment and one collection in order to remove any potential effects of cumulative collection. The use of 4 cohorts provided 4 individual plant replicates for each treatment and collection time. Grains were collected from the outside 2 florets (2nd and 4th floret) on the central eight spikelets of the ear of the main tillers, resulting in 16 grains being collected in total from each plant. All experimental research contained within this submission complied with institutional, national and international guidelines.

### Mature grain weight analysis

In order to determine the average weights of mature grains in Experiment 1, grains collected from primary heads were counted and weighed collectively. The total weight was then divided by the number of grains per head in order to give an average individual grain weight.

### Developing grain average dry weight and grain moisture content analysis

The 16 developing grains collected from each stage per plant in Experiment 2 were weighed before being frozen in liquid nitrogen and transferred to a − 80 °C freezer. Frozen grains were then freeze-dried for a 4-day period before being weighed again in order to determine the total combined grain dry weight. Average grain moisture, average grain dry weight and average percentage moisture content of grain were also calculated as follows: mc% = ((sample fwt – sample dwt)/fwt)*100, where *sample* fwt is the average fresh weight of a grain and *sample* dwt is the average grain weight of the same sample after drying.

### RNA extraction

Total RNA was extracted from freeze-dried material collected at 6, 10 and 14daa in Experiment 2, i.e. samples that had received 0, 4 and 8 days of temperature treatment respectively. Two extractions were performed on grain samples collected at 6daa, and three extractions were performed for each of the two treatments (high temperature/control) at the two further collection points (10 and 14daa) with 14 RNA extractions performed in total. Total RNA was extracted from samples via phenol/chloroform extraction as detailed in Singh et al. [69]. RNA purification and DNA removal was performed prior to resuspension of the RNA pellet using a Qiagen™ RNAse free DNase kit (79254).

### CDNA library preparation

The RNA samples were sent to the Earlham Institute (Norwich, UK) where they underwent additional testing, according to their quality control pipeline for RNA Sequencing. The integrity of the 14 RNA samples was analysed using an Agilent Bioanalyzer 2100™ before being confirmed as suitable to be run on an Illumina HiSeq 2500™ sequencer at a read metric of 2x125bp (paired-end reads) and with an expected output of 13–16 million reads per sample. Adapter dimer was removed from the most affected libraries before libraries were pooled together. The concentration of the pooled libraries was then validated via q-PCR which matched closely to concentrations obtained via Qubit. Therefore, in total, 14 samples proceeded to sequencing (2x6daa, 3x10daa (control), 3x10daa (treated), 3x14daa (control), 3x 14daa (treated).

### RNA-Seq data analysis

RNA-Seq reads from grain samples were mapped against the *T.aestivum* genome using the TGACv1 gene assembly produced by the Earlham Institute [70]. Read data underwent fastqc [71], and trimmomatic [72], in order to remove adapters before undergoing gene alignment using the alignment program for mapping HISAT2, [73]. RNA-Seq data from existing studies by Pearce et al. [23] and Pellny (*published online*: Arrayexpress - E-MTAB-8397), were also uploaded to the galaxy platform used as reference data sets for comparison.

Following mapping of the reads obtained from the RNA-Seq analysis, the number of reads that were aligned uniquely to each gene within the genome were counted using the featurecounts programme [74] in the R data analysis package [75]. The data were then normalised to account for variation in sequencing depth using the edgeR Bioconductor software package [76] and log counts per million (Log-CPM) was used as the measure of gene expression [77].

Comparative analysis between RNA-Seq samples was performed using the Rstudio statistical software package [75] using the advanced linear model function to identify genes that were differentially expressed between treated and control samples at the different developmental stages. All differential gene expression analysis was performed using a false discovery rate (FDR) of 0.05. The number of differentially expressed genes (DEGs) was identified between the three sampled developmental stages independent of treatment using pairwise comparisons also via*.* The edgeR statistical programme [76]. PCA analysis was performed using the DESeq2 Bioconductor software package [78].

### Tissue specific expression

In order to determine gene expression specific to an individual grain tissue, the RNA-Seq data collected from RNA extracted from whole grains were compared with data sets relative to specific grain tissues. Filtering was initially performed on the Pearce et al. [23] dataset by selecting DEGs with an expression value higher than 5 counts per million (CPM) in the tissue with the highest expression and an 8 log-fold change between the pericarp and endosperm. The list of pericarp predominant DEGs were also compared with RNA-Seq data obtained from dissected pure starchy endosperm collected at 17daa by Pellny, (*published online*: Arrayexpress - E-MTAB-8397) in order to confirm that none of the genes determined to be predominantly expressed in the pericarp were also expressed in endosperm. DEGs determined to be from the pericarp that had a measure of expression of more than 40 reads in 80 million in the starchy endosperm data list were assumed to be non-specific to the pericarp and were therefore discarded. One hundred seventy-five DEGs from the list of pericarp predominant DEGs met this threshold, reducing the number of pericarp predominant DEGs from 638 to 463.

In order to visualise patterns of gene expression, relative expression heat-maps were produced using z-score scaling and global clustering in order to group genes with similar expression profiles (Figs. 4, 5, 6). To scale via ‘z-scores’, read counts for a gene from the samples had the mean calculated and then subtracted from each sample score for that gene. The standard deviation was then calculated before each data point was divided by this standard deviation.

### K-means cluster analysis

CPM values were collected for each gene from the pericarp and endosperm predominant list of DEGs from the RNA-Seq analysis performed in this experiment and log transformed. The median of the log-CPM values for each of the five clusters from both the pericarp and endosperm were calculated in each sample and this value was used as measure for gene clusters.

### Gene ontology analysis

Functional analysis of the genes within each cluster were performed using the Knetminer™ web tool developed by the Bioinformatics department of Rothamsted Research Institute [79]. GO term enrichment using Blast2GO™ [80] was also performed on identified gene clusters. In the analysis, the reference genome used in the RNA-Seq analysis of this experiment (TGAC_v1.) [71] was compared against the genes within determined clusters using Fischer’s exact test.

### Statistical analysis

Grain weight and moisture content statistical analyses were performed using Genstat™ 17th edition [81]. Data were analysed via ANOVA: to analyse controlled environment material a blocking structure of cohort/cabinet/position of the cohort within the cabinet/position of the pot within the cabinet position/pot number was used. Comparison of gene cluster average CPM values was completed using a statistical analysis add-on in Microsoft Excel and selecting a 2-way ANOVA to compare clusters between treatments and developmental stages. Significant differences between results were determined by obtaining the least significant differences (LSDs) of the means between data and comparing this against the sample means. Where the difference between compared sample means was greater than the LSD value of the means, this was taken to indicate a significant difference (*p* = < 0.05) and vice versa with differences between samples means lower than the LSD value of the means.

## Additional Files

**Additional File 1.** Table 1S Grain yield data.

**Additional File 2.** RNA alignment rate.

**Additional File 3.** Table showing pericarp predominantly expressed genes arranged in clusters.

**Additional File 4.** Table showing endosperm predominantly expressed genes arranged in clusters.

**Additional File 5.** Diagram of experimental design.

## Data Availability

The datasets supporting the conclusions of this article are available in the ArrayExpress repository (www.ebi.ac.uk/arrayexpress) under the following accession numbers: Experiment data - E-MTAB-8520, Pellny data - E-MTAB-8397, Pearce data - E-MTAB-3103. In addition, the datasets supporting the conclusions of this article are included within the article (and its additional files).

## Abbreviations

CPM: Counts per million
Daa: Days after anthesis
DEGs: Differentially expressed genes
EXPs: Expansins
FDR: False discovery rate
Log-CPM: Log counts per million
LSDs: Least significant differences
LTP: Lipid transfer proteins
nsLTPs: Non-specific lipid transfer proteins
P.a: Post-anthesis
PAR: Photosynthetically active radiation
PCA: Principal component analysis
RH: Relative humidity
ROS: Radical oxygen species
XTH: Xyloglucan endotransglucosylase/hydrolases
