# Supplementary figures and images for: High post-anthesis temperature effects on bread wheat (Triticum aestivum L.) grain transcriptome during early grain-filling
Source: BMC Plant Biol. 2020 Apr 16;20:170. doi: 10.1186/s12870-020-02375-7 (PMC7164299; doi:10.1186/s12870-020-02375-7)

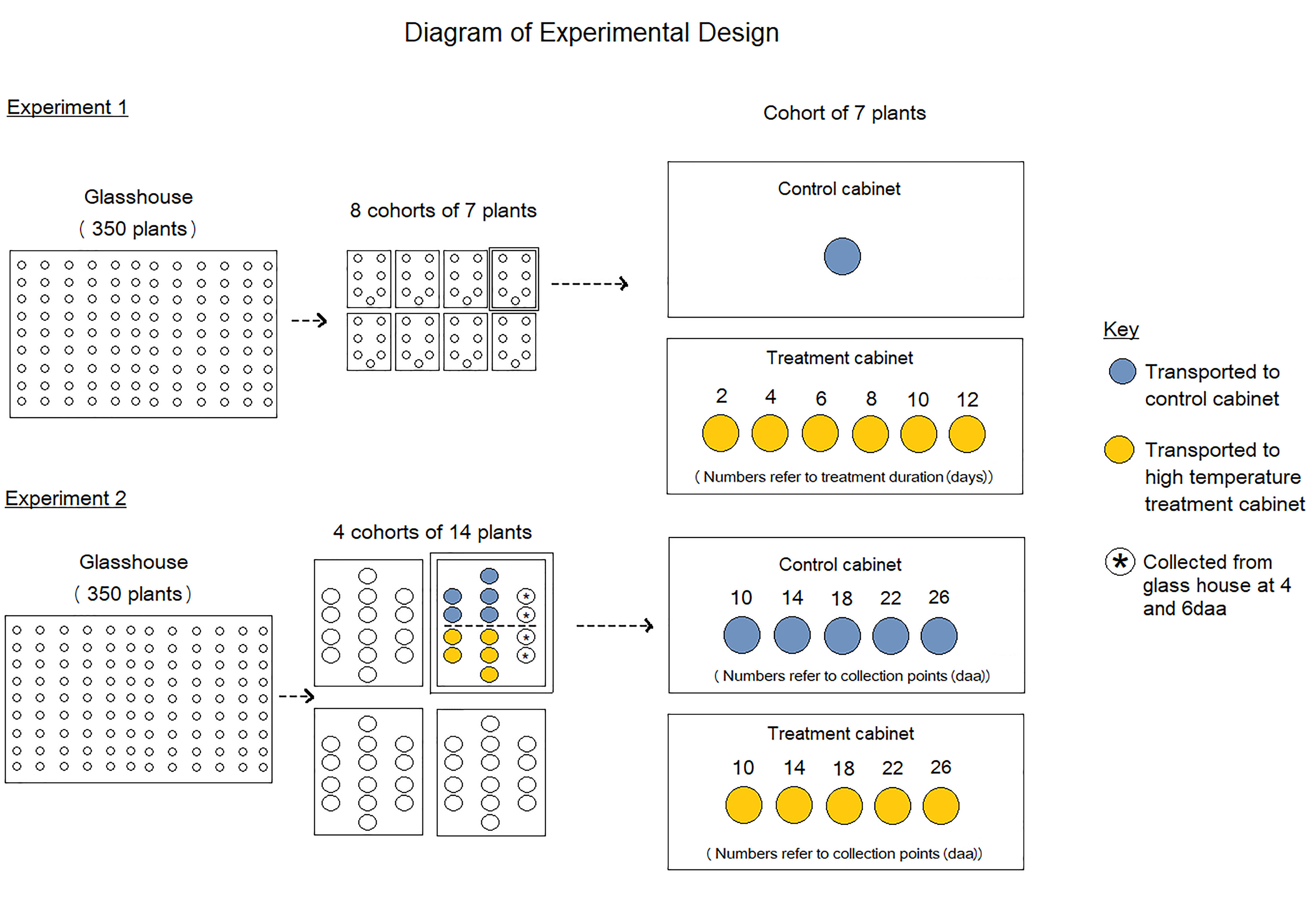

Supplement: Supplementary file 5 — Additional File 5. Diagram of experimental design. [file 12870_2020_2375_MOESM5_ESM.tif]
