# Supplementary material for: High post-anthesis temperature effects on bread wheat (Triticum aestivum L.) grain transcriptome during early grain-filling
Source: BMC Plant Biol. 2020 Apr 16;20:170. doi: 10.1186/s12870-020-02375-7 (PMC7164299; doi:10.1186/s12870-020-02375-7)
Supplement: Supplementary file 6 [file 12870_2020_2375_MOESM2_ESM.odt]

| Sample id | Raw reads | Filtered Reads | Overall alignment rate (%) |
| --- | --- | --- | --- |
| 6daa_1 | 6,654,796 | 6,611,383 | 90.76 |
| 6daa_2 | 16,212,825 | 16,019,024 | 86.18 |
| 10daa_S1 | 69,824,687 | 69,091,046 | 92.77 |
| 10daa_S2 | 11,867,987 | 11,751,171 | 89.66 |
| 10daa_S3 | 11,560,437 | 11,388,192 | 86.49 |
| 14daa_S1 | 8,064,011 | 7,897,691 | 90.60 |
| 14daa_S2 | 12,008,425 | 11,882,894 | 92.69 |
| 14daa_S3 | 6,223,486 | 6,195,569 | 90.65 |
| 10daa_C1 | 6,698,890 | 6,660,264 | 92.35 |
| 10daa_C2 | 5,976,952 | 5,946,620 | 90.31 |
| 10daa_C3 | 6,878,379 | 6,842,927 | 89.44 |
| 14daa_C1 | 4,650,052 | 4,544,391 | 90.71 |
| 14daa_C2 | 24,286,842 | 23,926,252 | 93.81 |
| 14daa_C3 | 5,175,351 | 5,085,325 | 79.10 |

Table 5.1 RNA quality scores and RNA-Seq statistics for 14 sequenced samples. RIN=RNA integrity number, S1, S2…=Stress sample, C1,C2…=Control sample. Raw reads = Number of 125BP paired end reads, filtered reads =Number of pure reads filtered for adapters and bad reads overall alignment rate = Percentage of filtered reads that were successfully aligned to the reference genome.
